# The Emerging Scenario of the Gut–Brain Axis: The Therapeutic Actions of the New Actor Kefir against Neurodegenerative Diseases

**DOI:** 10.3390/antiox10111845

**Published:** 2021-11-20

**Authors:** Thiago M. C. Pereira, Larissa Z. Côco, Alyne M. M. Ton, Silvana S. Meyrelles, Manuel Campos-Toimil, Bianca P. Campagnaro, Elisardo C. Vasquez

**Affiliations:** 1 Laboratory of Translational Physiology and Pharmacology, Pharmaceutical Sciences Graduate Program, Vila Velha University, Vila Velha 29102-770, ES, Brazil; pereiratmc@gmail.com (T.M.C.P.); larissazambom.coco@gmail.com (L.Z.C.); alyneton@gmail.com (A.M.M.T.); bianca.campagnaro@uvv.br (B.P.C.); 2 Federal Institute of Education, Science and Technology (IFES), Vila Velha 29106-010, ES, Brazil; 3 Laboratory of Translational Physiology, Physiological Sciences Graduate Program, Health Sciences Center, Federal University of Espirito Santo, Vitoria 29075-910, ES, Brazil; silvana.meyrelles@ufes.br; 4 Physiology and Pharmacology of Chronic Diseases (FIFAEC), Molecular Medicine and Chronic Diseases Centre (CIMUS), University of Santiago de Compostela, 15782 Santiago de Compostela, Spain; manuel.campos@usc.es

**Keywords:** oxidative stress, inflammation, neurodegenerative diseases, kefir, chronic focal encephalitis, angiotensin II, autonomic nervous system, gut microbiota

## Abstract

The fact that millions of people worldwide suffer from Alzheimer’s disease (AD) or Parkinson’s disease (PD), the two most prevalent neurodegenerative diseases (NDs), has been a permanent challenge to science. New tools were developed over the past two decades and were immediately incorporated into routines in many laboratories, but the most valuable scientific contribution was the “waking up” of the gut microbiota. Disturbances in the gut microbiota, such as an imbalance in the beneficial/pathogenic effects and a decrease in diversity, can result in the passage of undesired chemicals and cells to the systemic circulation. Recently, the potential effect of probiotics on restoring/preserving the microbiota was also evaluated regarding important metabolite and vitamin production, pathogen exclusion, immune system maturation, and intestinal mucosal barrier integrity. Therefore, the focus of the present review is to discuss the available data and conclude what has been accomplished over the past two decades. This perspective fosters program development of the next steps that are necessary to obtain confirmation through clinical trials on the magnitude of the effects of kefir in large samples.

## 1. Introduction

Neurodegenerative diseases (ND) are characterized by the slow and gradual degeneration of axons and neurons of different areas of the central nervous system (CNS), resulting in movement and/or cognitive disorders. These complex disorders are closely associated with oxidative stress and inflammation, which are two main systemic conditions that aggravate neurodegeneration [1,2]. This review is focused on the discussion of the therapeutic benefits of the probiotic kefir on oxidative stress and inflammation, which are important contributors to chronic neural disturbances, including Parkinson’s disease (PD), Alzheimer’s disease (AD), dementia, and epilepsy. 

Despite the continuous search for effective multidisciplinary approaches and neuroprotective therapies [3,4], ND are yet incurable and the patients suffering from these diseases succumb to the high medical costs. Based on the current data, it is estimated that 50 million people worldwide are affected by these disorders, mainly in low and middle-income countries [5]. It is estimated that a new case appears every 4 s, and forecasts point out that in 2050, the number of people suffering from these diseases will reach 115.4 million due to population aging [6].

## 2. Gut Microbiota: An Extraordinary Army of Fighters to Defend the Host

**Figure 1:**
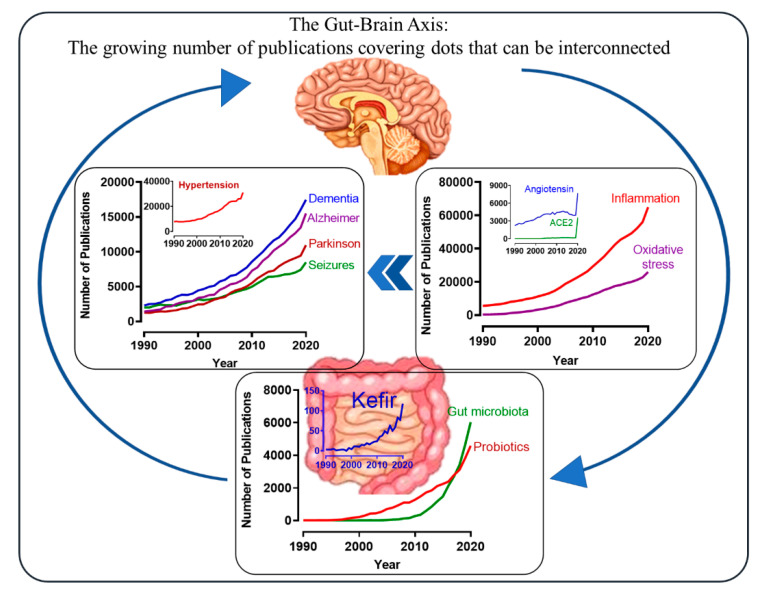
Schematic illustration and graphs constructed by plotting the number of papers published per year in indexed journals listed on the PubMed platform. The arrows indicate the multifactorial and interactive connections, including inflammation, oxidative stress, hypertension and angiotensin/ACE2. We interpreted the sharp increase in the number of published papers relating to angiotensin/ACE2 in 2020 as being due to its role in COVID-19. The bottom panel shows a large jump in the number of papers in “microbiota” (~26,000), demonstrating the current relevance of therapies based on the use of probiotics and that kefir shows a similar profile. ACE2: Angiotensin-Converting Enzyme type 2. The above diagram was created by one of the coauthors of this review, a 76-year-old researcher suffering from PD for 12 years.

Over the past 10 years, an extraordinary increase in the number of experimental and clinical studies aiming to understand the interactions among microbes inhabiting the host’s gut was observed [6,7,8]. As noted in the line graphs of Figure 1, which were constructed based on the number of papers that were published, they are indexed on the PubMed platform. The bottom graph of Figure 1 shows that the gut microbiota is 20 years old, i.e., as demonstrated by the few papers published in 2000 and the number of papers that were published in 2020. The line that indicates the evolution of papers related to probiotics has a similar slope and magnitude. The reason for this growing number of papers in this area could be because many investigators working with “omics-based” approaches have joined or created new opportunities in the field of microbiota [6,7,8,9]. Therefore, biomedical sciences are facing new opportunities and perspectives for new attractive scenarios.

**Figure 2:**
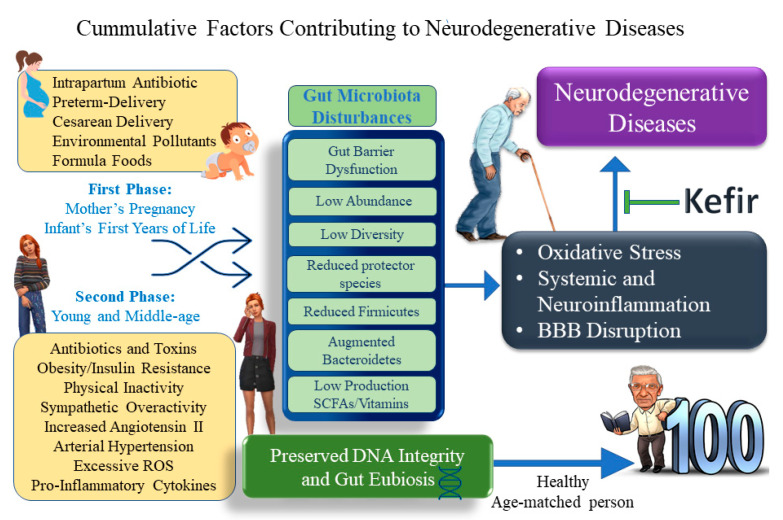
Schematic diagram suggesting several negative factors (conventional and nonconventional) that influence the seeding of the gut microbiota (as identified by “omics” approaches) in the early days of life of the infant and that can anticipate the onset of AD and PD. In addition to searching for the negative factors influencing ND, in our opinion, we suggest that the search in this field should also be expanded to the features (biomarkers, epigenetic influence) that could explain those that are centenarian life spans. This diagram was also created by one of the coauthors of this review, a 76-year-old researcher suffering from PD for 12 years.

Based on genetic sequence analysis of samples of meconium and feces [10], the general opinion is that the gut microbiota starts in the newborn under the influence of the health conditions of the prenatal mother’s health, gestational age, mode of delivery, type of feeding, quality of environment and exposure to toxins (see Figure 2, the schematic time course of evolution of the gut microbiota from infants to elderly people) [10,11,12,13,14]. Therefore, once the appropriate composition was established, the symbiotic community could be considered an incredible legion of 10^9^ to 10^12^ warriors composed of five main phyla.

The first role of the gut microbiota is to protect the host against pathogenic microorganisms and to neutralize the effects of toxins and/or drugs [12,13,14]. The second role is to provide essential metabolites/vitamins, facilitating the absorption of ions and molecules and thus generating an additional source of energy for colonocytes [15]. For example, dietary components absorbed through different segments of the small gut provide short-chain fatty acids (SCFAs) and contribute to reducing food intake, increasing energy expenditure, and improving insulin sensitivity (see elsewhere for details [16,17]). The third phase is related to the trophic process (growth and differentiation) of epithelial cell lineages, which are important for the development and maturation of the immunological system initiated in fetal life [18].

Considering that aging-related diseases can be consequences of changes during the early years of life (Figure 2), the analysis of the microbiota in infants has become an interesting issue [14,19]. It is important to emphasize that inadequate microbial colonization during this period can lead to dysbiosis, impacting health in adulthood. As shown in Figure 2, maternal disturbances such as hypertension, preeclampsia, obesity, and preterm delivery will affect the seeding of the newborn’s gut microbiota. A healthy pregnancy supports the development of the immune system of the fetus during the first years of life [13,20], which is important because the development of the immune system is a key condition for long-term survival. In this regard, there is a consensus that the aging process, associated with DNA damage, significantly influences the development and severity of ND. Although the meconium of newborns already shows an abundance of phyla and a great diversity of species, it is necessary to establish reference guides of normal values for generating medical reports [19]. As illustrated in Figure 2, the composition of the gut microbiota in the newborn can be influenced by sequential factors: mode of birth (vaginal vs. cesarean), type of nutrition (formula vs. breastfeeding), and environmental exposure to contaminants (e.g., bisphenol A) [14,20,21] and toxins [22,23]. In addition, it is known that during this stage of life, the maturation of gut-associated lymphoid tissue (GALT) occurs [24].

During infanthood, the gut microbiota is an interface mediator between internal and external factors since the genetic sequencing approach shows a diversified composition of mutualistic bacteria and yeasts [19]. These microorganisms protect the host and are associated with the achievement of immune homeostasis at maturity, which is known as “eubiosis”. In contrast, the term “dysbiosis” is associated with an imbalance between protective and pathogenic microorganisms, which makes the host susceptible to diverse invaders, providing nonhealthy metabolites and diverse immune outcomes [25]. Therefore, the goals of the present review are to discuss possible mechanisms by which the aging process can lead to ND and, in other cases, protect the person for more than one century.

Recently, epidemiological studies estimated that in 2050, the world population older than 60 years will increase 100% [26], which means that the high number of aging-related neurodegenerative and cardiometabolic diseases could also grow in the same proportion [27]. In many countries, people of all ages have been influenced by globalization and, unfortunately, abandoned the millenary “healthy diet” (e.g., the Mediterranean, and Asian diet pyramids; see the Book of Genesis 25:8 in the Bible) for the unhealthy Western-type diet [28,29,30], which is considered to be a negative factor. It is well established that a balanced microbiota is dependent on good dietary intake (rich in fiber, vegetables, and fruits with the secretion of bile acids accompanied by reduced intake of L-carnitine, lipopolysaccharides (LPS), and animal fats). Some evidence demonstrates that the dietary habits of the host are determinants of oxidative stress and inflammation. More specifically, a low-protein diet was associated with high levels of Bifidobacterium and Lactobacillus species [31]. In this regard, low levels of Bacteroidetes and Clostridium maintain SCFAs compatible with a well-shaped and selective gut barrier (increasing mucus secretion with tight junction integrity), avoiding the consequent overweight state [32]. Interestingly, conditions of malnutrition lead to an imbalance of Bifidobacterium and Lactobacillus [32], facilitating the growth of facultative anaerobes contributing to dysbiosis [16,33]. In addition to diet, the gut microbiota is also considered to be highly vulnerable to aging, pollutants, hygiene, and other environmental factors, such as smoking, high alcohol intake, and/or sedentary life (as illustrated in Figure 2).

The microbiota varies along the gastrointestinal tract (small and large portions) [34]. However, the main metabolism of microbes occurs in the luminal portion of the large intestine, which validates the use of stool samples to analyze the composition/distribution of microbes through microbiological, biochemical, or genetic approaches. Although a “gold standard” signature of healthy gut microbiota does not exist, our group currently considers a richness > 400 species and a genera diversity > 7 as important indicators of gut stability. Therefore, due to great variation between subjects of different ethnicities, genders, ages, and physiological statuses, a more accurate index still needs to be established for a better analysis. Despite that challenge, we account for the presence of at least four protector species: *Lactobacillus* spp. (1 to 6%) [35], *Akkermansia muciniphila* (1 to 5%) [36], *Faecalbacterium prausnitzi* (5 to 12%) [37] and *Bifidobacterium* spp. (1 to 6%) [38]. Despite the finding of the great variability in the dominant phyla Firmicutes and Bacteroidetes in healthy individuals, the relative percentage could also be used as an index of abundance [10]. In clinical practice, the most relevant marker of gut eubiosis can be calculated by the abundance of Firmicutes and Bacteroidetes (F plus B), in the range of 85–95%, and their proportion (F/B) has an index of approximately >0.7 [5]. Another piece of evidence is that most elderly healthy individuals exhibit a proportion of Firmicutes/Bacteroidetes (~0.6) [39]. On the other hand, it is commonly observed that nonhealthy aging can result in dysbiosis [40,41].

To close this issue, it is important to recognize the relevance of morphofunctional gut integrity of the epithelial wall border covered by mucus to prevent the entrance of pathogens and undesired diet-derived chemicals from crossing to the systemic circulation. Other factors, such as inadequate protective mucin production, dendritic cell dysfunction, abnormal tight junctions (compromising the immune system), and decreased production of food-derived bioactive compounds that are fermented by gut microorganisms, are also typical phenotypes of dysbiosis [42].

## 3. Gut–Brain Axis: Extraordinary Advances and Opportunities to Improve the Treatment of Dysbiosis

The gut microbiota–brain arch reflex intercommunicates in a bidirectional way (via the direct autonomic nervous system, immunological and neuroendocrine routes), which is an important structure in the pathophysiology of neurodegenerative disorders such as AD, epilepsy, and PD, with the pathophysiology occurring mainly when the gut microbiota is dysfunctional [43,44,45]. The dominant linking appears to be through the vagus nerve, which is composed of approximately 80% afferent (sensory) and 20% efferent (motor) fibers connecting the visceral organs to the brain [44,45,46,47]. Additionally, known as the “crossroad of neuroimmune interactions” (see [43]), this nerve is an important sensor of changes in microbiota metabolites, transferring gut information to the central nervous system [48]. Overall, this system can integrate cognitive and motor functions, enabling the triggering of an adapted or inadequate response. New evidence indicates that there is a cholinergic anti-inflammatory pathway through vagal nerve fibers (through a vagus–vagal reflex), promoting the tight junction functional integrity between enterocytes and, therefore, avoiding the vulnerability of leaking gut elicited by dysbiosis [43,45]. On the other hand, inputs from external situations of stress (e.g., “fight or flight” reactions) involving the periaqueductal gray matter [49,50], amygdala, and hypothalamus can inhibit vagal nerve activity. The dysfunction of these structures can be due to dysbiosis, which compromises the gastrointestinal tract, triggering gastrointestinal disorders (e.g., irritable bowel syndrome and inflammatory bowel disease) and/or ND, as described above [45,51].

The influence of the microbiota on enteric–associated neurons transcends vagal nerve participation. We also need to highlight the important role played by the combination of glucocorticoids and catecholamines in gut homeostasis. Regarding glucocorticoids, there is evidence that demonstrates that gut microbiota can modulate the stress-related hormone corticosterone (cortisol in humans). For example, Sudo et al. [52] showed that probiotic supplementation reduced corticosterone in germ-free mice. Corroborating these data, hypercortisolism was observed in germ-free rodents [53]. However, it is still necessary to clarify the possible pathways involved in this complex double-way relationship that links the brain, gut microbiota, and adrenal cortex function, which are closely related [54,55]. In parallel, several recent studies have demonstrated that some species of bacteria can produce catecholamines (e.g., norepinephrine), which also contributes to sympathetic responsiveness [56,57]. Interestingly, in 2020, another group demonstrated a possible “microbiota–dependent” regulator in the gut that can activate the sympathetic pathway through a gut–brain circuit [58]. By acting through a complementary mechanism, it is also known that gut dysbiosis can be involved in renin–angiotensin system overactivation [59,60,61] and potentiating target cells that express alpha/beta receptors. Therefore, it appears to be reasonable that there is a “vicious cycle” between the gut and the autonomic nervous system [62].

**Figure 3:**
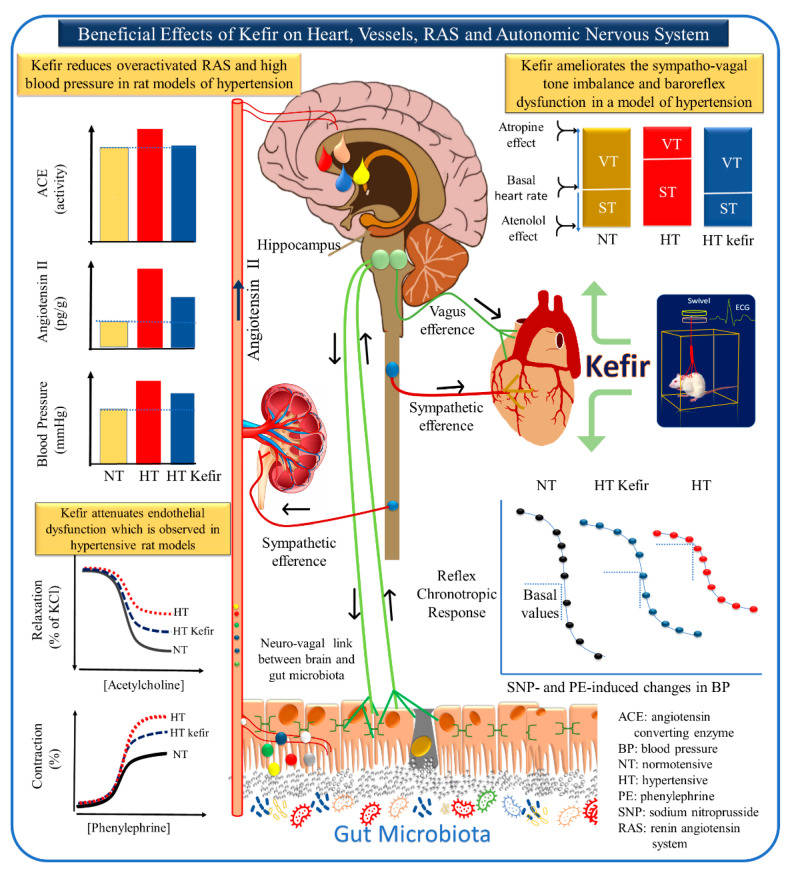
Gut microbiota and some relevant molecular pathways linking gut dysbiosis to cardiovascular diseases through the bloodstream and via the autonomic nervous system. The main mechanisms of the modulators include the neuroendocrine, afferent, and efferent pathways of the autonomic nervous system (vagus and sympathetic components), reactive oxygen species (ROS, represented by red circles), inflammatory markers (white and blue circles), and dietary metabolic products (short-chain fatty acids: SCFAs; lipopolysaccharide: LPS, green circles), which are delivered into the circulatory system and reach the brain, connecting integrative areas related to ND. Distinct areas (kidney, gut, heart, and brain) are represented to emphasize the protective effects of kefir based on our recent publications [14,63,64,65]. All images were modified and reconstructed from our previous publications. This diagram was also created by one of the coauthors of this review, a 76-year-old researcher suffering from PD for 12 years.

In recent years, we focused on cardiovascular/kidney diseases and probiotic supplementation using kefir. Although more details about kefir and its benefits on ND will be discussed in another section, it is important to show several direct/indirect sympatholytic mechanisms observed in our experimental investigations in mice and rats after exposure to beneficial bacteria (Figure 3).

## 4. The Brain–Heart–Kidney Interconnection: The Role of Kefir

The relationship between the brain and cardiovascular system has been a continuous focus of basic and clinical investigation. At the end of that century, the discovery of renin opened new avenues for the integrative relationship between the brain and the circulatory system. Here we revisited previous studies demonstrating integrative dots connecting the nervous system with the kidneys, heart, and blood vessels [47,64,66] and discuss its bidirectional systemic interactions (see Figure 3). Our hypothesis is that the prevalence of concomitant autonomic nervous dysautonomia, vascular dysfunction, and high blood pressure, is a pathophysiological condition, which when combined with cerebrovascular disorders can contribute or aggravate ongoing AD and other chronic or acute related ND. This review highlights the therapeutic actions of kefir on the overactivity of the renin–angiotensin system (RAS), the imbalance of the autonomic nervous system, and the attenuation of the endothelial dysfunction observed in different experimental models of hypertension [9,63,64].

After approximately 200 years of experimental and clinical research that led to the discovery of the RAS, angiotensin II is considered to be one of the most integrative endogenous substances in the human body [67,68,69,70,71,72]. RAS homeostatic actions are provided by kidney sensors for reduced sodium and volemia, leading to the production of renin into the venous blood and then synthesizing angiotensin I [69,70,71,72]. This peptide is converted to the bioactive octapeptide angiotensin II by an angiotensin-converting enzyme (ACE) that promotes hypertensive effects [73,74,75,76].

Currently, a growing body of evidence demonstrates an integrated association between RAS and the autonomic nervous system, which are important contributors to the etiology of hypertension (see Figure 1 and Figure 3) [50,77]. Approximately 30 years ago, our laboratory demonstrated in the angiotensin-dependent model of hypertension (2K1C) that the predominance of the vagal system controlling the heartbeat was overpassed by sympathetic activity, which also includes an increase in the cardiac rhythm and force [78]. Similar to other research groups, we were challenged to include, in our main research field, the integrative actions between the brain, renal, and cardiovascular systems, aiming to understand the etiology of primary and secondary hypertension [68,69,73,74]. Recently, we reported the relationship between the neural control of blood pressure and gut microbiota.

**Figure 4:**
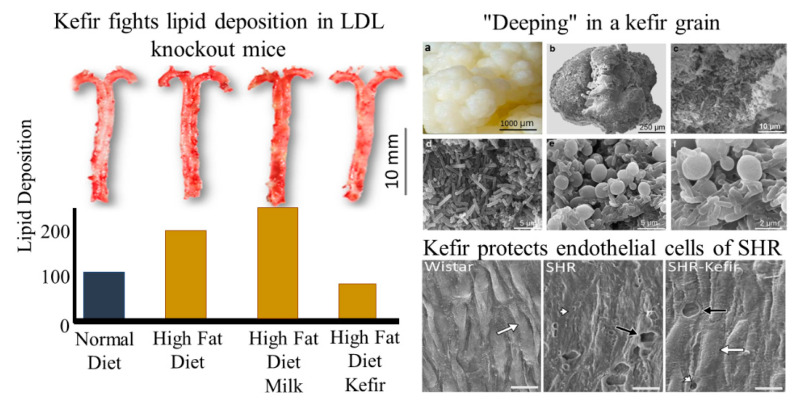
Illustrative images from our laboratory demonstrating the vascular protective role of kefir in both lipid deposition (in LDLr knockout mice) and in the endothelial layer (in SHR model). The images were reconstructed and modified from previous publications [7,80]. In addition, we demonstrate the “inside world” of a single grain, which was pictured at increasing magnifications (from “b” to “f”) and symbiotically inhabited by millions of microorganisms that our laboratory has used to demonstrate the effect of kefir experimental and clinical studies [7].

Our group and others have dedicated recent years to the investigation of the relationship between gut dysbiosis and hypertension [14,41,63,64,79,80]. Animal models (mice and rats) of essential and renovascular hypertension were used to investigate the effectiveness of probiotic kefir. In the spontaneously hypertensive rat (SHR) model, it was shown that chronic kefir administration (for eight weeks) caused a significant reduction of hypertension and improved endothelial dysfunction through the restoration of ROS/NO imbalance [7]. Later, this same group demonstrated in the same experimental model that this probiotic also protects against the effects of an environmental contaminant [14]. In addition, kefir showed similar effects to pharmacological ACE inhibitors [7,14]. Interestingly, this anti-ACE activity [65] and antiatherogenic effect (Figure 4) [80] can be achieved even using a soluble nonmicrobial fraction of kefir.

Monteiro and collaborators recently published a paper that demonstrates the antihypertensive actions of the probiotic kefir [63]. The authors clearly showed that kefir prevents high blood pressure, through renal and systemic RAS inhibition, in 2K1C hypertensive rats (see Figure 3). Other remarkable effects of kefir in this experimental model include the improvement of the nephron structure and endothelial dysfunction, attenuation of the high levels of ROS (plasma and kidney tissues), and the damaged architecture of the aortic endothelial surface [63].

For decades, we have observed in different models of hypertension that they share a common feature: the imbalance of the autonomic nervous system, mediated by angiotensin II in the nervous areas controlling blood volume by the paraventricular nucleus, subfornical organ, and hypothalamus [47,51,73]. Based on the above discussion, we can speculate that ND and cardiovascular diseases are linked to the microbiota–gut–brain axis, which can be the result of a malfunction of the vagal afferences and efferences. Using classical pharmacological approaches [73,81], our laboratory has demonstrated in renovascular hypertension, SHR, and even in the model of hypertension induced by the blockade of nitric oxide that the sympathetic nervous system is the main cause of sustained hypertension [82].

In 2015, two different research groups observed a possible link between gut dysbiosis and hypertension [83,84,85]. In subsequent years, several groups (including ourselves) have been deeply dedicated to the elucidation of this issue on how communication between gut microbiota and the sympathetic nervous system occurs. Klippel and collaborators [64] have provided new insights into the neural control of blood pressure and cardiac rhythm. They demonstrated that the cardiac vagal tonic control that characterizes Wistar-Kyoto (WKY) rats (the control of SHR) is overridden by sympathetic activity. Therefore, hypertensive individuals appear to share the same neural disturbances that trigger augmented vascular resistance and increased cardiac blood pumping to the aorta. The authors also used classical pharmacological approaches to investigate in the SHR a contribution of a possible beneficial effect of kefir on the brain-mediated reflex control of blood pressure. They found impaired vagal and sympathetic reflex control (baroreflex), which was significantly attenuated by treating the animals with the probiotic kefir for 60 days [64]. These results were also confirmed by spectral analysis of direct blood pressure recordings. Supporting our finding in the SHR, Toral, et al. [71] demonstrated in the same model increased sympathetic activity contributing to gut dysbiosis and reinforcing neuroinflammation, which compromises blood pressure control [71]. Altogether, tonic and reflex brain control of blood pressure suggest that probiotic kefir is a promising nonpharmacological therapy, at least in primary (SHR) and secondary (two-kidney, one clip (2K1C)) hypertension models. The possible applicability of this new therapy still needs to be confirmed in further clinical trials. Interestingly, neuronal abnormalities, such as dementia, AD, and obstructive sleep apnea, are also characterized by brain vascular anomalies, such as blood–brain barrier (BBB) disruption [40,85,86,87], which appears to be related to gut dysbiosis [86]. Probiotic supplementation could be a promising adjuvant strategy against the development of cardiovascular disease and ND (see Figure 3 and Figure 4).

## 5. Lessons on How to Fight Oxidative Stress

In 1985, when Sies and Cadenas characterized for the first time the terminology “oxidative stress in cells and organs”, few researchers or clinicians measured the real impact of this player on the course of several chronic NDs [88]. Over recent decades, this “free radical chemistry” has advanced beyond a subfield of biochemistry, reaching major interdisciplinarity in physiology, microbiology, pathology, and pharmacology. However, it is already known that oxidative stress is an important player in the pathogenesis of several NDs by acting in a “vicious cycle” that negatively impacts aging [40,87]. Classically, this global concept in redox biology and/or medical areas is defined as the state of imbalance between oxidants and antioxidants (favoring oxidants) leading to a disruption of redox signaling, molecular damage [88,89], and inflammation [90,91,92]. Recent data reveal that this redox balance is fundamental for quartet “gut-brain-microbiota-immune cells”, which modulate oxidative stress that is intimately involved in the breakdown of the gastrointestinal tract and BBB [93,94]. In this section, we summarize recent data that reveal robust interactions between oxidative stress and the “amazing world of warriors” fighting to maintain the integrity of the gut–brain axis.

It is known that the nervous system requires high energetic demands, enhancing exergonic oxidative processes and culminating in neurons often exposed to ROS such as superoxide anion (O_2_^•−^), hydrogen peroxide (H_2_O_2_), nitric oxide (NO) and their conversion into powerful oxidants, such as hydroxyl radical (•OH) and peroxynitrite ion (OONO^•−^) [40,89,95]. At the same time, several studies have demonstrated that the nervous system presents low levels of antioxidant enzymes such as superoxide dismutase (SOD) in neurons and glutathione/glutathione peroxidase (GSH/GPx) localized in astrocytes, as recently revised by us and others [87,96,97,98], which are susceptible to apoptosis by p53 signaling [99]. Therefore, these mechanisms appear to contribute to the vulnerability of the central nervous system, as elicited by oxidative stress and are susceptible to degenerative processes. More recently, studies have revealed that complex microbiota–host cross-talk could also play a modulatory role in oxidative stress in the central nervous system through direct and indirect (such as lipopolysaccharides, amyloid proteins, or antibiotics) pathways, which can reach the brain through circulation or the vagus nerve, activating microglia to overproduce ROS [87,100]. Thus, the microbiota–gut–brain axis has been an “open gate” for new therapeutic strategies for several neurological conditions, as described in the next sections.

Although physiological low oxidant exposure normally requires redox control and cell signaling, supraphysiological concentrations address unspecific targets and lead to the inhibition of mitochondrial functions and structural modification of lipids, proteins, carbohydrates, and not the least, damage to DNA [74,89,101,102,103]. For example, lipid peroxidation triggered by ROS culminates in crescent loss of membrane fluidity accompanied by an increase in permeability to Ca^2+^ and a membrane potential drop. In parallel, recent data found that under eubiosis conditions, the epithelial lining of the gut could also generate basal levels of ROS, contributing to the homeostasis of the gut barrier and indirectly protecting the central nervous system [87]. On the other hand, gut dysbiosis could be both a cause and a consequence of increased levels of ROS in the central nervous system and consequently contributes to pro-oxidative and proinflammatory mechanisms, leading to the neurodegenerative process [95,104,105].

Currently, oxidative stress is researched by traditional indirect methods and is also explored in neurodegenerative diseases by evaluating products of lipid peroxidation (e.g., malondialdehyde [106,107] and 4-hydroxynonenal [108]), products of oxidized proteins (e.g., advanced oxidation protein products (AOPP)) [40], and by “comet assay”, an efficient tool to measure DNA breaks at the single-cell level [101,103,109,110,111]. Furthermore, direct methods of detection were adopted using confocal and live-cell imaging, flow cytometry, and/or HPLC methods (for more details, see Dikalov et al., 2014 [112]), enabling the investigation of the distinct participation of O_2_^•−^, H_2_O_2_ and •OH/OONO^•−^ species [40,101,102,103,113,114,115,116,117,118]. Since these assays show high analytical sensitivity and precision, indirect and direct methods are complementary tools to investigate possible therapeutic strategies against neurodegenerative diseases, as discussed later.

In summary, in a century marked by pharmacological therapeutic failures despite unparalleled scientific advances, deepening the knowledge of the diversified pathways through which probiotics could exert a role in counteracting NDs appears to be of great relevance. Given the role of the gut microbiota in health and the prophylactic/therapeutic potential of probiotics, this review will focus on successful probiotic strategies against neurodegenerative abnormalities such as AD and PD and ND-epileptic disorders that manifest as repeated seizure episodes.

## 6. Probiotics in ND: Why Would It Be a Useful Tool for Drug Interactions?

Emerging studies have shown that the use of probiotics can provide an interesting strategy against the progression of ND, reducing neuroinflammation [40,119,120,121], ameliorating gastrointestinal function [122,123] and diminishing gut leakiness [124,125]. Drugs do not create any effect but instead modulate physiological functions. Therefore, strategies using probiotics, which improve the performance of the nervous system, can synergically optimize the drug response to maintain and/or to recover the drug effects in those patients generally allocated in the “refractory” groups. Some successful examples in different neurodegenerative diseases will be presented below.

Regarding AD, Akbari et al. (2016) [107] demonstrated (for the first time) that chronic probiotic consumption containing *Lactobacillus acidophilus*, *Lactobacillus casei*, *Bifidobacterium bifidum*, and *Lactobacillus fermentum* (2 × 10^9^ colony forming units CFU/g for each one) improved cognitive function and some oxidative and proinflammatory biomarkers. However, two years later, another study conducted by the same research group revealed inconsistent effects with patients with dementia, which was justified by some limitations (a common issue in this type of trial), such as a small number of subjects, the inclusion of patients mostly in the severe stage of AD, the dosage and formulation of probiotic bacteria, and a sort of supplement exposure time [126]. Although preliminary animal evidence supports the potential protective role of probiotics on cognitive function, a recent meta-analysis covering AD subjects is controversial, and further large-scale controlled trials with long-term, randomized trials are still needed [127].

The second example is about epileptic patients. Unfortunately, drug-resistant epilepsy is an associated problem that has higher morbimortality levels and low quality of life than the general population [128]. In parallel, inflammation is a mainstay in the pathophysiology of human epilepsy, and proinflammatory serum cytokines are associated with the severity and frequency of seizures [128,129,130]. Although many questions about the biomolecular mechanisms involved remain unanswered, Gómez-Eguílaz et al., (2018) [128] recently observed probiotic supplementation (*Lactobacillus acidophilus*, *Lactobacillus plantarum*, *Lactobacillus casei, Lactobacillus helveticus, Lactobacillus brevis, Bifidobacterium lactis, B. lactis, and Streptococcus salivarius* subsp. *Thermophilus,* CFU~10^11^ for each) could decrease the number of seizures and improve the quality of life of patients. Interestingly, only in three years was a positive effect of probiotic supplementation shown in an experimental model of seizures induced by pentylenetetrazole (PTZ) using *Lactobacillus rhamnosus*, *Lactobacillus reuteri*, and *Bifidobacterium infantis* (CFU~10^9^ for each, via gavage for 3 weeks) [131]. More recently, Kilinc et al. (2021) [132] corroborated the antiepileptic effect of prebiotic + probiotic supplementation in Wistar weaner rats (CFU~10^9^ containing *Bifidobacterium lactis*, *Bifidobacterium breve*, *Bifidobacterium longum*, *Bifidobacterium bifidum*, *Lactobacillus acidophilus*, *Lactobacillus casei*, *Lactobacillus plantarum*, *Lactobacillus salivarius*, *Lactobacillus rhamnosus*, *Lactobacillus bulgaricus*, *Lactobacillus paracasei*, *Streptococcus thermophilus*, *Ascophyllum nodosum* and inulin), also exhibiting antioxidative activity and alleviating neuroinflammation.

PD is a multisystemic disease characterized by impairments in motor system function associated with loss of dopaminergic neurons in the substantia nigra [133,134,135]. Motor impairment (characterized by resting tremor, postural instability, and muscle rigidity) and nonmotor symptoms (sensory disturbances, olfactory dysfunction, pain, and gastrointestinal dysfunction) have long been recognized as classical hallmarks of PD [135,136,137]. Recent reviews have revealed that modifications of the microbiome and several potential molecular mechanisms of the gut microbiota are linked to the pathogenesis of PD (see [135,137,138]). In 2011, the first clinical study demonstrated that PD patients with chronic constipation receiving fermented milk containing *Lactobacillus casei Shirota* for 5 weeks improved stool consistency and defecation habits [122]. Five years later, another study using probiotics (60 mg per tablet of *Lactobacillus acidophilus* and *Bifidobacterium infantis*) for 3 months also reduced bloating and abdominal pain in subjects with PD [139]. In 2019, a randomized, double-blind, placebo-controlled clinical trial using probiotic products (containing *Lactobacillus acidophilus*, *Bifidobacterium bifidum*, *Lactobacillus fermentum,* and *Lactobacillus reuteri*) was conducted to observe clinical (movement) and biochemical (metabolic parameters) outcomes in PD patients [140]. Interestingly, supplementation with this product for 3 months resulted in favorable impacts on malondialdehyde (MDA), blood glutathione (GSH) (reducing oxidative stress), insulin sensitivity, and diminished high-sensitivity C-reactive protein (hs-CRP). Although the potential exists to predict the beneficial effects of probiotics in PD, the mechanisms are still unclear, diverse, and broad. Moreover, experimental and/or clinical evidence that demonstrates benefits in PD is still very limited, and more studies for confirmation are required [137].

It is well established that levodopa (plus carbidopa or benserazide), a common dopamine-replacing substance, is the most effective drug used to control bradykinetic symptoms. However, it is known that the “on-off” motor fluctuations in patients with PD are highly dependent on levodopa bioavailability, which is indirectly modulated by dietary amino acids and gut microbiota [121]. Recently, studies from van Kessel et al. (2019) [141] highlighted that the abundance of bacterial tyrosine decarboxylase in the proximal small intestine (e.g., *Enterococcus*) could explain the increased dosage regimen of levodopa treatment in PD patients due to excessive early degradation of dopamine outside the brain. Probiotic supplementation could be an interesting strategy to alter gut microbiota composition, reduce neuroinflammation in both gut and brain sites, diminish gut leakiness, avoid bacterial translocation and improve gastrointestinal function [137,142]. Therefore, considering that probiotic supplementation in neurological patients (including refractory patients) could avoid therapeutic failure and reduce polypharmacy and/or toxicity (due to unnecessary readjustment), probiotic-drug interactions are a promising line of research and could have relevant implications for patients, their families, and person-centered care.

## 7. The Symbiome and Pathobiome: A New Understanding of the Gut in ND

Although the term “microbiome” has generally been used in the biomedical area, it is important to clarify that this word excludes any eukaryotes, whereas the term “symbiome” describes the whole assemblage of associated organisms, excluding the host [143]. Since in our review we are showing the relevance of prokaryotes in the luminal gut against ND, the “microbiome” term could be applied throughout the text. Even so, it must be considered that the probiotic kefir also contains fungal species such as *Kazachstania*, *Kluyveromyces*, *Naumovozyma* [144], *Saccharomyces cerevisiae*, *Kluyveromyces marxianus* (formerly Candida kefyr) [145] and *Candida albicans* [7], and we cannot exclude the beneficial involvement of these eukaryotic microorganisms in progressive neurodegenerative diseases.

In the same way, the term “pathobiome” refers to a set of host-associated organisms (viruses, prokaryotes, and eukaryotes) linked with decreased health status as a result of interactions in the host. Additionally, in this review, our emphasis is to demonstrate the impact of prokaryotes on dysbiosis. Therefore, there is an interest in researching the impact of viruses in the neurodegenerative process, such as herpes simplex virus type 1, cytomegalovirus, and varicella-zoster virus encephalomyelitis virus (see [146,147,148,149]).

## 8. Brief History and Biochemical Properties of Kefir: A Basis for Appropriate Supplementation

According to tradition, kefir grains (see Figure 4) were gifted by Allah to the Prophet Mohammed, who passed it to Caucasians who spread it worldwide, passing it hand-to-hand [150]. The origin of fermented milk goes back to antiquity, most likely when man began to use animal milk in his food. Caucasians discovered that fresh milk carried in leather bags (animal skins) could occasionally ferment, resulting in a fizzy drink [151] whose shelf life was longer than that of raw milk [152]. The Bible also describes a product similar to kefir, called “manna”, as a food that was miraculously produced, being provided by God to the Israelite people, led by Moses, during his stay in the desert toward the promised land (Exodus 16), which justifies the term “prophet’s drink” [153,154]. Kefir is fermented milk also known as tibicos, “the prophet Mohammed’s grain”, “Tibetan mushrooms”, “yogurt plants”, “yogurt mushrooms”, “kephir”, “kiaphur”, “kefer”, “knapon”, “kepiand”, and “kippi”. The term derives from the Turkish “keif”, which means “well-being” or “well-living” [100,153,154,155].

In some parts of the world, kefir is still not yet a popular product. However, in Central Europe, Asia, and some American countries, it has been commercially available and made on an artisanal scale for individual consumption [100,156] by the fermentation of different types of milk (see Table 1). Even so, this fermented milk gained adherents due to its functional properties. Kefir is a fermented, sour, slightly alcoholic milk produced from grains that contains a relatively stable population of microorganisms [7,157]. The fermentation process generates a series of compounds that impart a characteristic flavor and aroma to kefir, in addition to bioactive substances that are responsible for its nutraceutical properties [158]. Existing data suggest health benefits due to the regular consumption of kefir beverages. These compounds were associated with kefir’s biological properties, such as immunomodulatory [159], antimicrobial [160,161], antitumor [162], anti-inflammatory [40,163], and antioxidant [7,14,40,63,101,115] properties. These health-promoting benefits are associated with kefir microorganisms, the interactions between them, and the bioactive compounds produced from the fermentation of milk [164]. There is a symbiotic association of yeasts, lactic acid bacteria, and acetic acid bacteria, among other microorganisms [100,151]. However, the microbial composition of kefir can vary according to the region of origin, the time of fermentation, the type of substrate, and the manipulation techniques [7,165,166].

The prophylactic and therapeutic effects of lactic acid bacteria were studied at the beginning of the last century when Ilya Ivanovich Metchnikoff (the father of gerontology) launched the theory of prolonging life through regular consumption of fermented milk. Since then, scientists have corroborated these observations, relating the consumption of probiotic microorganisms with the modulation of disease status in various experimental models [7,63,101]. The most isolated microorganisms from kefir grains comprise the genera Lactobacillus (*L. casei*, *L. acidophilus*, *L. brevis*, *L. kefiri*, *L. plantarum*, *L. kefiranofaciens subsp. kefiranofaciens*, *L. kefiranofaciens subsp. kefirgranum*, *L. parakefir*), Lactococcus (*L. lactis subsp. lactis*), Leuconostoc (*L. mesenteroides*), Acetobacter, Kluyveromyces (*K. marxianus*), Saccharomyces [7,165,166] and eventually other genera listed above). Although this microbiota is in symbiotic equilibrium, it does not always remain constant [7,191,192].

The traditional method of kefir production occurs directly by adding 4% of the grains to the milk, preferably pasteurized or boiled, and then cooled to 25 °C (room temperature) for grain inoculation [7,9]. After the fermentation period, which varies from 18 to 24 h, at room temperature, the grains are separated from the fermented beverage by filtration and later used for inoculation in a new substrate. The filtrate submitted to lactic fermentation was transferred to the refrigerator and remained for 24 h. In this phase, the yeasts will produce alcohol and CO_2_, making the product more refreshing [7,101,193].

The double fermentation of milk by bacteria and yeasts results in a food rich in lactic, acetic, and glycolic acids, ethyl alcohol, CO_2_, vitamin B12, and polysaccharides, which give the product unique sensory characteristics [194]. The physicochemical composition of kefir varies considerably with the type of milk used in fermentation. A typical kefir contains 89–90% (m/m) moisture, 0.2% lipids, 3.0% protein, 6.0% carbohydrates, 0.7% ash and 1% alcohol and lactic acid [195,196]. To summarize, the organoleptic characteristics of kefir could be due to its main end-products [197]. For example, ethanol and CO_2_ impart a unique and exotic refreshing aroma to kefir [193]. Additionally, lactic acid promotes a slightly acidic and bitter taste, and acetaldehyde is related to the characteristic flavor of fermented milk [198].

Currently, we are facing a growing number of studies that are focused on isolated probiotic components. On the other hand, our laboratory faces the future of kefir without ignoring history, since we use traditional whole milk fermented from kefir grains in our experimental and clinical studies. An advantage of studying a kefir grain is that it is a high spectrum resembling a whole microbiome. Likewise, in kefir grains, the polysaccharide kefiran (also present in other milk-fermented products) serves as a matrix where bacteria and yeast live and proliferate [199,200]. Although the metabolic pathways that lead to the production of kefiran are not well understood, this polysaccharide is composed of repetitive units of hexa- and heptasaccharides that consist mainly of glucose and galactose, with branches [37,192]. To preserve and explore the richness of kefir grains, our group has taken advantage of the synbiotic characteristics of kefir (for a detailed description, see [150]), simultaneously exploring the probiotic (bacteria plus yeasts) and prebiotic (kefiran) components.

## 9. Targeting the Near Future: Milk Kefir against Oxidative Stress and Inflammation

Strong evidence supports the medicinal applications of kefir. In general, kefir acts on the intestinal microbiota, mediating protective/therapeutic effects through its probiotic microorganisms and/or bioactive compounds [201]. In this regard, kefir improves the host’s health by providing compounds that will systemically reach target organs and brain integrative areas such as those related to AD and PD [201,202]. These potentially beneficial compounds were yielded during the fermentation process, including lactic and acetic acids, vitamins, volatile compounds, nutraceutical components, and, especially, small peptides derived from milk proteins, including “captopril-like effects” [9,164]. Recently, many studies have reported the important role of kefir, most of which associate bioactive compounds with the antioxidant and/or anti-inflammatory properties of beverages [163,182,203,204,205].

As mentioned above, oxidative stress causes severe damage to biological systems, leading to the development of chronic diseases. In this context, evidence points to the antioxidant properties of kefir. In vitro, kefir showed antioxidant potential measured by DPPH free radicals and ABTS assays [177]. In addition to its antioxidant actions, kefiran showed dose-dependent protection of proteins from oxidative injury [173,206], and kefir also demonstrated significant antioxidant effects in vivo. Over the past decade, our group has shown that kefir is a likely food for the treatment of oxidative stress-dependent diseases [7,14,40,63,80,101,115]. Kefir intake decreases the production of ROS [7,101] and increases antioxidant enzyme (catalase, superoxide dismutase, and glutathione-peroxidase) activity [207,208,209]. Therefore, kefir safeguards the cells from the harmful effects of ROS by protecting proteins, lipids, carbohydrates, and nucleic acids, avoiding the apoptosis process [7,40,101,115,210].

Previous evidence points to the anti-inflammatory and immunomodulatory potential of kefir. Complications associated with inflammation are a key cause of morbidity and mortality due to chronic diseases. Peptides from kefir inhibited the NF-κB signaling pathway [163], increased anti-inflammatory (IL-10), and decreased the production of proinflammatory cytokines (such as INF, IL-1β, IL-6, and TNF) [40,115,159,204,211].

In summary, the beneficial effects of kefir are associated with its anti-inflammatory and antioxidant properties, as previously demonstrated by us and others [40,163,212], preventing apoptosis and, consequently, neuronal degeneration [213,214]. Therefore, this probiotic beverage shows the potential to act as an adjuvant in conventional therapies addressing ND.

## 10. ND and Kefir: Promise or Reality?

NDs are chronic incurable, debilitating conditions characterized by movement and cognitive disorders due to progressive neuronal dysfunction and disability associated with oxidative stress and inflammation, which are two main systemic conditions that aggravate neurodegeneration [1,2]. It is estimated that a new case appears every 4 s, and forecasts point out that in 2050, the number of people suffering from these diseases will reach 115.4 million due to population aging [5].

Knowledge about the structure of the nervous system and the molecular processes underlying the functioning of neuronal cells is crucial to understanding the pathophysiology and molecular damage in ND [98]. Despite different etiologies, oxidative stress and inflammation are common features of ND since they are associated with neuronal cell death and shrinkage in specific brain areas [98,215]. Neuronal stress is associated with synaptic dysfunction, impaired protein degradation systems, increased ROS production, mitochondrial dysfunction, DNA damage, inflammation, and excitotoxicity by mechanisms including cAMP dependence [98,216,217,218,219,220].

Neurodegeneration has been associated with inflammation and its mediators, which together lead to endothelial dysfunction (leading to BBB disruption) [44], apoptosis [221], necroptosis, [222,223], neuronal autophagy [224,225], and astrogliopathy [226] and accumulation of Aβ and tau protein [227,228]. These events associated with ROS trigger neurodegenerative events [227,229,230,231]. In addition, other pathways were explored. As an example, in 2012, for the first time was described by Iliff et al. [232] an important mechanism of brain metabolic waste clearance called as “glymphatic system” (which stands for glial-dependent lymphatic transport) [233]. There is experimental evidence that accumulation of Aβ and tau protein could occur in the brain due to impaired glymphatic clearance, aggravating the ND [228,232,233]. Among the proteins involved in this system, AQP4 seems to play a key role in the brain fluid homeostasis and justifies, at least in part, the failure of the glymphatic system [234,235]. Although it is an exciting area in research, innovative diagnostic and therapeutic strategies involving this issue are still necessary, since glymphatic system in the human brain needs to be characterized in more detail [228].

Beyond endogenous factors, the environment can influence disease risk and course, contributing to the pathogenesis of ND [236,237]. The type and composition of diet during life have important long-lasting effects on brain function [238]. In addition to its known effects in cardiovascular diseases, nutrients induce epigenetic changes in neurons, which are associated with degenerative disorders (see Figure 1) [239,240].

In recent years, an increase in the number of papers linking changes in the gut microbiota to ND was observed (see Figure 1). The microbial composition of the gastrointestinal tract influences neuronal tissue through various pathways, such as immune, neurological, and endocrine signaling [241,242], affecting behavior, BBB integrity, neurogenesis, and neurotransmitter production [243]. In response to oxidative stress, gut microbiota diversity is altered, which could even trigger neuroinflammation and, consequently, neurodegeneration [244,245].

Regarding this issue, modern setups (e.g., “intestine-on-chip”, organoids, and 3D cultures) would be necessary to monitor the effects of kefir and molecules involved in the gut–brain axis [246,247].

In this last section of the review, we will provide for the first time a review highlighting the findings that were published in the last 3 years emphasizing the neuroprotective properties of kefir, which is the object of investigation by our translational research group in Brazil.

### 10.1. What Is New in Dementia?

Dementia in AD is a progressive, global, and irreversible decline in cognitive functions mainly in elderly patients [227,248]. AD shows characteristics of disseminated neurodegeneration and two classical etiopathogenic biomarkers: neuritic plaques (NPs) and neurofibrillary tangles (NFTs) [98,227,249]. NPs are extracellular deposits of β-amyloid peptides, whereas NFTs are formed by the aggregation of hyperphosphorylated tau protein [227,250,251]. The neurodegeneration process in AD is a dynamic and multifaceted biochemical phenomenon. The presence of soluble amyloid β oligomers (AβOs) induces synaptic dysfunction due to aberrant activation of N-methyl D-aspartate (NMDA) receptors and abnormal increases in postsynaptic Ca^2+^ levels, leading to excitotoxicity [227,248]. In addition, tau hyperphosphorylation could be the necessary point between dysfunction and neural death [227,252].

According to oxidative stress theory, neuronal death in AD occurs due to ROS that interacts with cellular biomolecules, causing functional changes that precede cardinal neuropathological manifestations of this disease [40,227,249,252]. Experimental studies have demonstrated that due to high ROS generation accompanied by a low level of antioxidant compounds, cell regeneration is an early and auto-limited phenomenon before apoptosis and the formation of senile plaques and NFTs [227,252,253].

Neuroinflammation is a process that plays a relevant role in the pathogenesis of AD [254]. Previous evidence has identified an increasing number of proinflammatory molecules involved in the cognitive impairment of AD, such as interleukin (IL)-6, tumor necrosis factor-alpha (TNFa), and the inflammasome complex (NLPR3) [255,256,257]. In addition, other authors have demonstrated positive associations between proinflammatory cytokines (e.g., IL-1, IL-6, TNF-α, IL-8, and IL-12) and AD progression [40,254,258]. Furthermore, these neuroinflammatory cytokines can compromise β-amyloid clearance, leading to the accumulation of this protein in the brain [254,259,260].

The classical scenario that prevailed for several decades highlighting oxidative stress and inflammation as pivotal mediators in the pathogenesis of AD was recently joined by an ascending and multifaceted “amazing actor”: the gut microbiota (see Figure 1). However, the latter actor does not shine alone (as in a monologue) but acts in an intense interaction (dialog) between oxidative stress and inflammation. As an example, bacterial lipopolysaccharides can increase the levels of cytokines and other proinflammatory molecules, which are directly associated with AD [261,262,263]. On the other hand, different sources of probiotic supplementation can modulate cognitive processes of learning and memory [40,264,265,266,267], reducing oxidative stress [40,252,268] and proinflammatory cytokine levels [40,254].

For the first time, data from our group published by Ton et al., 2020 [40] demonstrated that supplementation with probiotic kefir for 90 days brings substantial improvements in global cognitive function and immediate and late memory and a significant improvement in functions involving constructive skills. In addition, kefir reduced ROS, leading to an attenuation of plasma protein oxidation, proinflammatory cytokines, and apoptosis in AD patients [40]. Although the metabolic and hemodynamic profiles were not evaluated in our study, it is well known that the chronic use of probiotics favorably modifies the cognitive capacity of subjects with dementia, in addition to improving blood pressure, insulin sensitivity, and lipid profile [107,269].

It is important to emphasize that other studies have demonstrated that kefir supplementation also contributes to the neuromodulatory process that mediates neuroactive and neuroendocrine syntheses (involving, for example, acetylcholine, dopamine, serotonin, noradrenaline, adrenaline, glutamate, gamma-aminobutyric acid and brain-derived neurotrophic factor (BDNF)) and the expression of its receptors [35,107,270,271,272,273].

### 10.2. Encephalitis and Kefir: A New Insight

Encephalitis is characterized by inflammation of the brain tissue due to direct infection or an autoimmune response and is recognized as a common refractory illness [274]. Among them, Rasmussen encephalitis (RE) is a rare chronic inflammatory ND, defined by progressive and diffuse brain inflammation/deterioration (and consequently unilateral brain atrophy), with significant cognitive decline and hemiparesis and, unfortunately, intractable epilepsy [115,275,276,277]. Although the pathophysiology is still unclear, multifocal inflammation, immune-mediated gliosis restricted to the cerebral hemisphere [277,278], microglial activation [279] and, more recently, dysbiosis was observed [115].

To date, the available treatments with antiepileptic drugs and hemispherectomy present incipient results in the control/reduction of seizures [280]. At the same time, previous studies have related dysbiosis with increased release of inflammatory cytokines and increased neuronal excitatory activity, especially in the hippocampal area [115,281], and these factors are considered to trigger the processes of epileptogenesis and neuroinflammation [282,283]. In this context, modulation of the intestinal microbiota could be a therapeutic strategy for epilepsy [284]. Among the functional foods that could beneficially alter the intestinal microbiota are probiotics, including kefir, which has shown positive results, such as the ability to restore the composition of the intestinal microbiota in individuals with autoimmune diseases [35,115].

RE has an etiology and pathophysiology that has not yet been explained, but the number of studies relating neurodegenerative diseases, including epilepsy, associated with dysbiosis has increased in recent years [283,285]. In the gut microbiota, there are more than 500 species of microorganisms [286,287]. In this regard, the effects of probiotic foods on the evolution and development of different diseases have been increasingly investigated [7,35,63,101,115]. Among the most studied microorganisms are the genera Lactobacillus and Bifidobacterium, which produce lactic, acetic, and propionic acids, reduce intestinal pH, produce bacteriocins, and produce biosurfactants, exerting microbial antagonism [288].

Inflammatory cytokines are biomarkers associated with brain inflammation in patients with epilepsy [289]. Recently, Hermann et al., 2001 [290] showed that TNF-α presents neuromodulatory properties that alter neuronal excitability. Confirming these data, RE patients treated with an anti-TNF-α drug (adalimumab) showed a reduction in epileptic seizures [291]. In addition, Kobylarek et al., 2019 [289] demonstrated that IL-1B levels are associated with generalized clonic-tonic epileptic seizures, IL-6 with the severity of the seizures, and IL-8 with partial seizures and severity.

Although the mechanism of action of kefir in the gut–brain axis is not fully understood, clinical evidence suggests that the reduction of dysbiosis represents a possibility of adjuvant treatment for refractory epilepsy, a condition that impacts not only the quality of life but also cognitive and motor functions [115]. Therefore, the screening of nonconventional therapeutic strategies aiming to control seizures appears to be a rising strategy to fight against neurodegenerative diseases. For example, the gut maintenance of Lactobacillus and Bifidobacterium was associated with the ability to attenuate serum levels of inflammatory markers such as IL-1B and TNFα [115,292,293]. On this subject, in the current year, our group published findings in Lemos et al. (2021) [115], which demonstrated (for the first time) a significant increase in the number of *Bifidobacterium* spp. and *Lactobacillus* spp., in an RE patient, suggesting that kefir can treat dysbiosis by modifying the colonization of the gut microbiota. Moreover, the probiotic kefir had a possible neuroprotective effect due to the modulation of the microbiota, which was associated with reduced expression of inflammatory cytokines and ROS production, resulting in less cognitive damage [115]. The increase in these genera of bacteria proved to be an important indicator of gut microbiome reestablishment [250,294,295].

### 10.3. Fighting PD with Kefir: Current Scenario and Future Horizons

**Figure 5:**
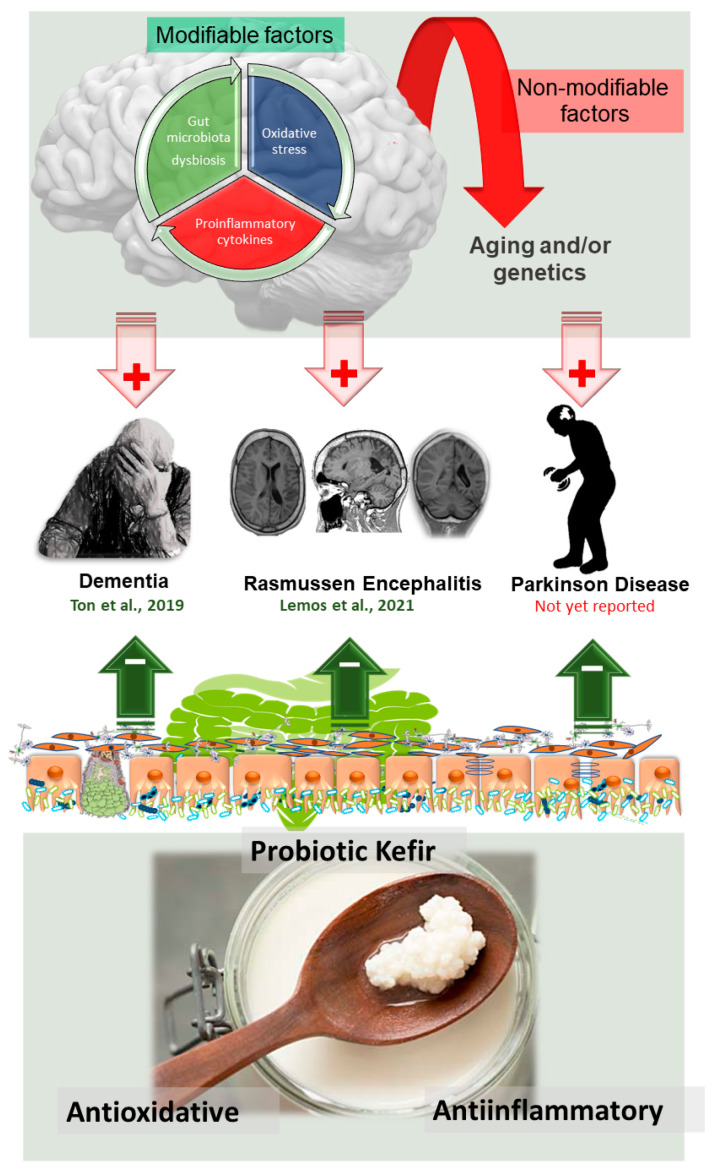
Graphical scheme demonstrating the state-of-the-art and our conclusive opinion based on our clinical studies performed in patients suffering from aging-related dementia and AD and in young patients exhibiting repeated and intense episodes of seizures. It should be noted that the role of kefir in PD progression has not yet been reported in the literature.

Although the consumption of fermented milk has been linked to health and longevity [296], its association with PD still needs more investigation. Recently, Olsson et al. (2020) [296] published a large cohort study that included approximately 82,000 Swedish adults, and the results confirmed the association between milk intake and an increased risk of PD (as previously observed by others) [297,298]. In contrast, fermented milk (soured milk and yogurt) intake was not associated with an increased risk of developing PD [298], opening a timely urgency to test new “stars” among probiotics (but with discovery millenniums ago). To date, as noted in Figure 5, trials involving kefir have not yet been published. Notably, the triad “inflammation-oxidative stress-neurotoxic processes” is involved in PD. On the other hand, kefir supplementation can attenuate these related pillars, as previously observed in clinical investigations published for members by our research group (abovementioned). Thus, this probiotic, which exhibits features of synbiotic fermented milk (probiotics and prebiotics that beneficially affect the host, see [299]), must become a potential therapeutic strategy against PD progression in future years.

## 11. Conclusions and Perspectives for Future Research Advances

Cardiovascular disease and ND can significantly undermine the quality of life of an individual, which indicates the need for scientific research that seeks to discover possible alternatives for treatment or even the improvement of the patients’ quality of life. In this review, we showed the current landscape and the future horizons of probiotic kefir in chronic diseases, aiming to translate its effects into real-life outcomes, mainly in cardiovascular disease and ND. The opportunities in the field of kefir as a nonpharmacological intervention for chronic diseases stem to a great degree from what we can learn about how it influences the gut microbiota and interacts with the host. The gut microbiota plays a key role in the pathogenesis of cardiovascular disease and ND by influencing the pro-oxidant and proinflammatory status. Although these endpoints have not currently been completely met, we discuss recent insights and promising results from the perspective of possible therapeutic applications of this probiotic. This perspective emerged in recent years when kefir research was driven by the characterization of microorganisms as well as by the postbiotic compounds found in beverages. For this circumstance, in vitro, in vivo, and in silico approaches were designed to uncover the effects of kefir on chronic diseases. Understanding the influence of individual differences on clinical outcomes would greatly contribute to the efficacy of kefir supplementation in chronic diseases. However, elucidating the interactions between the intestinal microbiota and kefir continues to present a challenge. In this regard, future research must focus on the stratification of clinical trials based on individual characteristics, including microbiota composition.

## Figures and Tables

**Table 1:** Chemical compounds that have been founded in the main animal milk sources used in kefir fermented process, as has been published by different authors.

Chemical Compound	Origin and Amounty of Kefir Animal Milk (Measurement Data)				Biological Activity
	Cow	Goat	Sheep	Camel	
Ash	0.7% [165] 1.1% [167] 1.2 % [168] 0.5 % (*w*/*v*) [152]	0.62% *w*/*w* [169]	0.94g [170]		Inorganic residue
Carbohydrates and Lactose	6.0% [165] 5.0 % (*w*/*v*) [152]	4.6 % (Lactose) [171] 5.9% *w*/*w* [169]	5% (Lactose) [171] 2.92 g (Lactose) [170] 4.11 g [170]		Basic nutrient
Grain Exopolysaccharide	2 g/L Kefiran [172] No measurements have been reported about kefiran concentration in the fermented kefir				Antihypertensive [173] Immunomodulation [40,174] Antitumoral [175] Antimicrobial [160] Antioxidant [176]
Fats	0.2% [165] 2.3% *w*/*v* [166] 1.3 % *w*/*v* [152]	3.4% *w*/*v* [171] 2.6% *w*/*w* [169]	5.3% *w*/*v* [171] 5.21 g [170]		Basic nutrient
Vitamin A and Carotene	B- Carotene 0.005 mg/100 g [177]		B- carotene 0.004 mg/100 g [177]		Antioxidant [177]
Vitamin E	0.025 mg/100 g [177]		0.04 mg/100 g [177]		Antioxidant [177]
Vitamin B1	10 mg/Kg [168]				Basic nutrient
Vitamin B2	5 mg/Kg [168]				Basic nutrient
Vitamin B5	3 mg/Kg [168]				Basic nutrient
Protein	3.0% [165] 3.9% *w*/*v* [166] 3.1% *w*/*v* [152]	3.45% *w*/*v* [171]	4.1% *w*/*v* [171] 5.71 g [170]		Basic nutrient
Total Nitrogen (%)	0.21 [178]	0.25 [178]	0.24 [178]	0.23 [178]	
Bioactive Peptides	AVPYPQR [9] Kefir peptides powder—KEFPEP [179,180,181,182,183]	LEIVPK [184] ARHPHPHLSFM [185] LGPVRGPFP [185] TAQVTSTEV [185] VLNENLLR [185]	(Antihypertensive) DKIHPF [170] (Antioxidant) QEPVLGPVRGPFP [170]		“Captopril like effect” [9,184,185] Antihypertensive [170] Antioxidant [9,170,181,185] Lipid metabolism modulation [180] Antifibrotic [181] Antithrombotic [185] Anti-inflammatory [179,181,182] Prevent menopausal osteoporosis [183] Prevent non-alcoholic fatty liver disease [179] Antidepressant [181] Antibacterial [185]
Aminoacids (threonine, serine, alanine, lysine, valine, isoleucine, methionine, phenylalanine and tryptophan)	Ranging 60–376 mg/100 g [168,186,187] 6–46 mg/kg [178]	4–52 mg/ kg [178]	1–26 mg/kg [178]	7–56 mg/kg [178]	Basic nutrient
Phenolic compounds	67 mg/100mL [177]	50–70mg/100mL [188]	78 mg/100mL [177]		Antioxidant [189,190]
Lactic acid	1.0% [165] 1.4–17.4 mg/mL [166] 0.6% *w*/*v* [152]	7.1 g/L [171]	9.1 g/L [171]		
Alcohol	0.48% [165] 0.5mg/mL [166]	1% [191] 9.5 ug/g [171]	12.5 ug/g [171]		
CO_2_	201–277 mL/L [165]				
Potassium	1.65% [168] 1009 ppm [178]	159 mg/100 g [169] 1049 ppm [178]	11.2 mg/L [170] 88.3 ppm [178]	1477 ppm [178]	Basic nutrient
Calcium	0.86% [168] 0.22% [166] 88 ppm [178]	116 mg/100 g [169] 132 ppm [178]	1765 mg/L [170] 37 ppm [178]	154 ppm [178]	Basic nutrient
Phosphorus	1.45% [168] 733 ppm [178]	735 ppm [178]	458 ppm [178]	783 ppm [178]	Basic nutrient
Magnesium	0.3% [168] 100.3 ppm [178]	10.3 mg/100 g [169] 203 ppm [178]	206 mg/L [170] 116.7 ppm [178]	83.4 ppm [178]	Basic nutrient
Sodium	15.6 ppm [178]	17.1 ppm [178]	20.73 ppm [178]	20.83 ppm [178]	Basic nutrient
Copper	7.32 mg/Kg [168] 0.06 ppm [178]	0.08 ppm [178]	0.02 ppm [178]	0.01 ppm [178]	Basic nutrient
Zinc	92.7 mg/Kg [168] 2.17 ppm [178]	0.34 mg/100 g [169] 2.23 ppm [178]	0.55 ppm [178]	2.43 ppm [178]	Basic nutrient
Iron	20.3 mg/Kg [168] 4.73 ppm [178]	2.25 ppm [178]	3.53 ppm [178]	4.86 ppm [178]	Basic nutrient
Manganese	13 mg/Kg [168] 0.25 ppm [178]	0.27 ppm [178]	0.21 ppm [178]	0.19 ppm [178]	Basic nutrient
Cobalt	0.16 mg/Kg [168]				Basic nutrient
Molybdenum	0.33 mg/Kg [168] 0 ppm [178]	0.01 ppm [178]	0.03 ppm [178]	0 ppm [178]	Basic nutrient
pH	4.54 [178]	4.6 [178]	4.51 [178]	4.1 [178]	
